# Incidence and Relative Survival of Patients with Merkel Cell Carcinoma in North Rhine-Westphalia, Germany, 2008–2021

**DOI:** 10.3390/cancers16112158

**Published:** 2024-06-06

**Authors:** Andreas Stang, Lennart Möller, Ina Wellmann, Kevin Claaßen, Hiltraud Kajüter, Selma Ugurel, Jürgen C. Becker

**Affiliations:** 1 Institute of Medical Informatics, Biometry and Epidemiology, University Hospital Essen, Hufelandstr. 55, 45147 Essen, Germany; 2 School of Public Health, Department of Epidemiology, Boston University, 715 Albany St, Boston, MA 02118, USA; 3 Cancer Registry of North Rhine-Westphalia, Gesundheitscampus 10, 44801 Bochum, Germany; lennart.moeller@stud.uni-due.de (L.M.); ina.wellmann@krebsregister.nrw.de (I.W.); kevin.claassen@krebsregister.nrw.de (K.C.); hiltraud.kajueter@krebsregister.nrw.de (H.K.); 4 Department of Dermatology, University Hospital Essen, Hufelandstr. 55, 45147 Essen, Germany; selma.ugurel@uk-essen.de (S.U.); j.becker@dkfz-heidelberg.de (J.C.B.); 5 German Cancer Consortium (DKTK), Partner Site Essen/Düsseldorf, Hufelandstr. 55, 45147 Essen, Germany; 6 Translational Skin Cancer Research, University Medicine Essen, Hufelandstr. 55, 45147 Essen, Germany; 7 Faculty of Biology, University of Duisburg-Essen, Universitätsstrasse S05 T05 B24, 45117 Essen, Germany

**Keywords:** (MeSH) carcinoma, Merkel cell, incidence, survival analysis, registries, Germany

## Abstract

**Simple Summary:**

Due to the rarity of Merkel cell carcinoma (MCC), only a few studies on both incidence and survival have been conducted. We provide up-to-date population-based incidence and relative survival estimates of MCC. We analyzed data from the cancer registry of North Rhine-Westphalia, Germany, for the years of 2008–2021, covering a population of 18 million individuals. We included all newly diagnosed MCC cases and calculated incidence rates and relative survival (observed divided by expected survival). Our analysis included 2164 MCC patients. The incidence of MCC was 5.2 and 3.8 per million for men and women, respectively. The 5-year relative survival was 59% in men and 71% in women. Survival was lower among men than women in all age–sex groups and was the highest for MCC of the upper extremity in both men and women. In terms of survival, the first two years are particularly critical.

**Abstract:**

Background: To date, only a few population-representative studies have been carried out on the rare Merkel cell carcinoma (MCC). We provide incidence and survival estimates of MCC, including the conditional relative survival. Methods: We analyzed data from the cancer registry of North Rhine-Westphalia, Germany, 2008–2021, covering a population of 18 million. We included all newly diagnosed MCCs and calculated age-standardized (old European Standard population) incidence rates and unconditional and conditional relative survival. Results: Our analysis included 2164 MCC patients. The age-standardized incidence of MCC was 5.2 (men) and 3.8 (women) per million person-years. The 5-year relative survival was 58.8% (men) and 70.7% (women). Survival was lower among men than women in all age–sex groups and was highest for MCC of the upper extremity in both men (68.2%) and women (79.3%). The sex difference in survival is particularly due to the better survival of women with MCC of the head and neck. In terms of survival, the first two years are particularly critical. Conclusions: Our data validate the worse survival among men and highlights a more favorable prognosis for MCCs located on the limbs. The first two years after diagnosis of MCC are the years with the highest excess mortality.

## 1. Introduction

Merkel cell carcinoma (MCC) is a rare neuroendocrine cutaneous malignancy that was first described in 1972 [1]. MCC is highly aggressive, and patients with MCC have a considerably lower survival probability than patients with cutaneous melanoma. In a recent analysis of 1.4 million newly diagnosed skin cancer patients in England spanning 2013–2019, the 5-year overall survival probability was 80.2% for cutaneous malignant melanoma and 40.9% for Merkel cell carcinoma [2]. Risk factors for MCC are older age, immunosuppression, pre-existing hematologic neoplasms, chronic UV exposure, and thus a history of other cutaneous tumors. MCC can occur due to two distinct etiologies: virus-associated etiologies, caused by clonal integration of Merkel cell polyomavirus (MCPyV), or virus-negative driven etiologies, caused by UV-induced DNA mutations and damage [3].

Treating MCC typically involves a combination of therapies, depending on the stage and extent of the disease. Surgical excision is the standard treatment for localized MCC tumors. A wide local excision with clear margins together using a sentinel lymph node biopsy is recommended to remove the primary tumor and check if the cancer has spread to nearby lymph nodes. Radiation therapy targeting the tumor bed and the draining lymph node region is frequently used as an adjuvant treatment. More recently, adjuvant immunotherapy with immune checkpoint inhibitors (ICIs) has been shown to improve progression-free survival [4]. Moreover, ICIs have revolutionized the treatment landscape for advanced MCC. Indeed, immunotherapy has become the preferred first-line treatment for advanced MCC due to its improved efficacy and durability of responses compared with chemotherapy [5].

Several countries showed an increase in MCC incidence over time in non-Hispanic whites, and a latitude closer to the equator was found to be associated with MCC incidence in North American men, but only to a small extent in women, possibly due to occupational sun exposure patterns [6]. Due to the rarity of MCC, population-representative relative survival studies quantifying the excess mortality due to MCC and its therapeutic consequences are scarce [7]. Five-year relative survival estimates from population-based registries are available from a few populations, including the United States (1973–1999 and 1986–2004) [8,9], the Netherlands (1993–2007 and 1993–2015) [10,11], Finland (1983–2004) [12], Spain (1994–2002) [13], New Zealand (2000–2015) [14], Germany (2007–2011) [15], and Queensland (1993–2010) [16].

Here, we analyzed data from the cancer registry of North Rhine-Westphalia (NRW), Germany. A population-based cancer registry is able to validly determine the incidence and survival for a clearly defined population, whereas clinical registries of individual university hospitals do not have a clear population reference. However, population-based cancer registries often have the disadvantage that data on the clinical phenotype of the cancer and on details of the therapy as well as certain outcomes (e.g., progression-free survival, PFS) are only incompletely available.

The aim of this study was threefold. First, we provide the most recent population-based incidence rates of MCC. Second, we present population-based, up-to-date 5-year relative survival estimates by sex, age, and anatomic location. Third, in order to provide reliable data for the design and interpretation of adjuvant and neoadjuvant trials [4], we explore the conditional survival of MCC, that is, the survival of MCC patients who survived the first, second, third, and fourth year after diagnosis of MCC.

## 2. Material and Methods

The cancer registry of North Rhine-Westphalia (NRW) is one of the largest European population-based cancer registries, which covers 18 million people, of which 21% are older than 65 years and 11% are older than 75 years. Statewide cancer registration started in 2005.

Cancer reporting in NRW is mandatory and is empirically dominated by pathology reports. The estimated completeness of cancer registration overall is >90% since 2008 [17]. We extracted all incident primary Merkel cell carcinoma cases (*International Classification of Diseases for Oncology, 3rd Edition* (ICD-O) [18], M8247/3) registered by the Cancer Registry of NRW in the years 2008–2021. The vast majority of the 2164 cases were coded as MCC of the skin (ICD-O topography codes C44.0-C44.9) with the exception of *n* = 1 (external upper lip, C00.0), *n* = 1 (overlapping lesion of lip, C00.8), *n* = 1 (base of tongue, C01.9), *n* = 3 (parotid gland, C07.9), *n* = 1 (nasal cavity, C30.0), *n* = 1 (connective, subcutaneous and other soft tissue of lower limb and hip, C49.2), *n* = 1 (labium minus, C51.1), *n* = 2 (vulva, not other specified, C51.9), *n* = 1 (prepuce, penis, C60.0), *n* = 1 (glans penis, C60.1), *n* = 1 (scrotum, C63.2), and *n* = 3 (unknown primary site, C80.9). The low number of MCCs of unknown primary relates to the application of the ICD-O rules. The ICD-O rules, which are used worldwide in population-based cancer registries, stipulate that a reported morphology code M8247/3 (Merkel cell carcinoma) is assigned the topography C44 if a localization is missing unless another localization is explicitly reported.

We grouped the following anatomic locations: head and neck (C44.0-C44.4, C00.0, C00.8, C00.2, C01.9, C02.1, C07.9, C30.0), face and ears (C44.0-C44.3, C00.0, C00.8), scalp and neck (C44.4), trunk (C44.5), upper limb including shoulder (C44.6), lower limb including hip (C44.7, C49.2), genitals, overlapping, and unknown anatomic sites (C51.1, C51.9, C60.0, C60.1, C63.2, C44.8, C44.9, C80.9).

Patients with complete TNM (tumor, nodes, metastases) staging data were categorized according to the UICC (Union Internationale Contre le Cancer) as stages I–IV [19]. If information on T, N, or M stage was missing, a categorization into at least UICC I–II or UICC III–IV was performed from the available information if possible. For example, if a tumor was only known to be N0 and M0 with a missing T-stage, this tumor was classified as UICC I–II. If it was only known that a tumor was N+ (i.e., N1-N3) with a missing M-stage, this tumor was classified as UICC III–IV.

### Statistical Methods

Through the use of the annual population figures by age and sex, we calculated age-specific and age-standardized incidence rates for the overall registration period by sex. We used the “old” European Standard Population [20] for the age standardization of incidence rates. For future comparisons of incidence rates in Germany with other countries, we also provide age-standardized incidence rates, standardized with the world standard population and the U.S. 2000 standard population, in the Supplementary Materials.

We calculated the relative survival, which is defined as the ratio of the observed probability of survival and the expected probability of survival. In principle, the observed survival probability by Kaplan–Meier estimation in a calendar year of an age and sex group is divided by the expected survival probability in that calendar year generated from the general population life table for the same age and sex group [21]. A relative survival probability below 100% indicates excess mortality due to the cancer or its sequelae. The advantage of relative survival compared with disease-specific survival is that the cause of death (death certificates), which is often incorrectly stated, is not required for relative survival. For the 5-year cumulative survival probability, the five annual relative survival probabilities are multiplied together [22]. Survival estimates were calculated with the R package periodR [23]. The cancer registry obtains information on vital status through record linkage with all death certificates of people in North Rhine-Westphalia. The last complete linkage was carried out as of 31 December 2021. This date was used as the closing data for the follow-up. We also estimated conditional relative survival of patients who survived the first, second, third, and fourth year after diagnosis of MCC. We stratified survival estimates by sex, age (<65, 65–74, 75–84, 85+ years), and topography.

A period analysis methodology was employed to derive the most up-to-date relative survival estimates [24,25,26]. The period analysis approach used available survival observations during the calendar period of 2017–2021 of the patients diagnosed during 2012–2021 (i.e., in addition to those diagnosed in the period of 2017–2021). Patients who survived until 2017 contributed (left truncated) survival experience to the analysis as well. Supplementary Figure S1 illustrates the data usage.

Due to missing data on stage (UICC), we performed multiple imputation using the assumption of missing at random to replace missing values. Statistical details of the multiple imputation can be found in the Supplementary Materials. We calculate and report the standard errors (SE) or 95% confidence intervals (95%CIs) to assess the precision of our estimates, as our goal is estimation and not significance testing. We wish to avoid publication bias by preferential reporting of significant results. Instead, we judge the value of our estimates by their precision and validity [27,28].

For the study of the association between sex and overall survival and disease-specific survival, we ran Cox proportional hazards regression models and used different degrees of adjustment. The first model only included sex. The second model additionally included age and topography. The third model additionally included age, topography, and UICC stage. We included these adjustment variables because they were associated with sex and were predictors of the outcome. In the complete case analysis, the proportional hazards assumption was checked by Schoenfeld residuals [29]. The proportional hazards assumption was not violated.

## 3. Results

During the period of 2008–2021, a total of 2164 patients (1049 men, 1115 women) with newly diagnosed MCC were registered. Tumors were found in the following localizations: head and neck, 891 cases (41%); trunk, 177 cases (8%); upper limb, 512 cases (24%); lower limb, 297 cases (14%); and other, unspecified locations, or genital skin, 287 cases (13%). The median age at diagnosis was 77 years (25th percentile [P25]: 70, 75th percentile [P75]: 82 years) and 79 years (P25: 72, P75: 85 years) among men and women, respectively. The age-standardized incidence rate of MCC during 2008–2021 was 5.2 and 3.8 per million person-years for men and women, respectively. This means that the age-standardized incidence rate is 35.3% (95%CI 23.5–48.2%) higher for men than for women. The age-specific incidence rates were higher for men in each age group. The age-standardized incidence rate of MCC was highest in the skin of the head and neck among both men and women (Table 1 and Table S1).

Because the UICC tumor staging is based on clinical parameters (e.g., tumor diameter), which are sometimes collected only during the course (e.g., status of the sentinel lymph node), and because the registration of incident Merkel cell carcinoma cases is based primarily on reports from pathologists, the UICC tumor stage was missing in 59% and 62% of male and female cases, respectively. A higher proportion of missing UICC values was associated with older age at diagnosis, localization (especially missing localization and skin of the genitals), early years of diagnosis, and patient death, especially deaths not attributable to MCC (e.g., death due to myocardial infarction, etc.) (Table S2).

**Figure 1:**
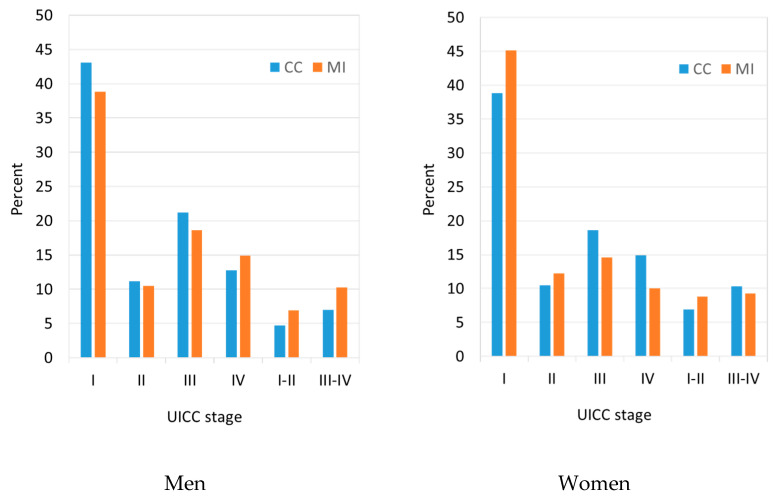
Distribution of UICC stages among 2164 patients with newly diagnosed Merkel cell carcinoma in North Rhine-Westphalia, Germany, 2008–2021, before and after multiple imputation. CC: Stage distribution according to complete case analysis; percentage distribution only among cases with UICC staging information. MI: Stage distribution after multiple imputation of missing data.

In the 854 patients for which the UICC stage was reported, the stage distribution for males and females was as follows: stage I—43.1% and 50.8%, stage II—11.2% and 12.0%, stage I/II—4.7% and 4.5%, stage III—21.2% and 17.9%, stage IV—12.8% and 8.0%, and stage III/IV—7.0% and 6.8%, respectively. Even after multiple imputation, men had a less favorable UICC stage distribution than women (Figure 1).

The 5-year relative survival (5yr-RS) was 58.8% in men (95%CI 52.3–65.4%) and 70.7% in women (95%CI 64.2–77.3%). The 5yr-RS was markedly lower in men than in women in each age stratum (<65, 65–74, 75–84, 85+ years). The 5yr-RS for head and neck MCC was 54.0% (95%CI: 41.8–66.1%) and 72.5% (95%CI: 62.4–82.6%) in men and women, respectively. The 5yr-RS was highest for MCC of the arms in women (79.3%, 95%CI 65.8–92.8%) and men (68.2%, 95%CI 55.8–80.6%) (Table 2). Both the complete case survival analysis, which had only patients without missing data for UICC, and the survival analysis, which was carried out after multiple imputation, showed that, after adjustment for age, topography, and UICC, men still had a less favorable prognosis (overall mortality and disease-specific mortality) than women (Table 3).

**Figure 2:**
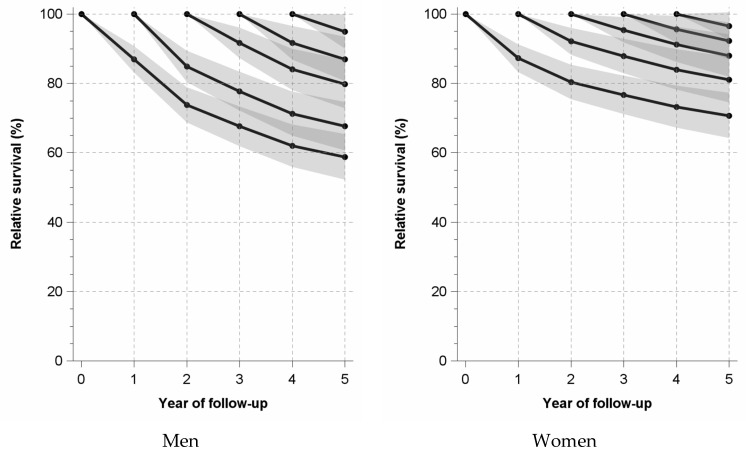
Unconditional and conditional relative survival (%) among men and women with newly diagnosed Merkel cell carcinoma in North Rhine-Westphalia, Germany, 2017–2021. Legend for Figure 1: Unconditional relative survival (period approach) starts at year zero of follow-up. Conditional relative survival starts after 1, 2, 3, and 4 years, respectively.

Unconditional relative survival, which is determined from the time of diagnosis, shows the sharpest drop of relative survival probability for both sexes within the first year after diagnosis (men: −13.0 percentage points; women: −12.7 percentage points). Even in the second year after diagnosis, there is still a marked drop in the relative survival probability, particularly for men (men: −13.2 percentage points; women: −6.9 percentage points). In the following years, the percentage point drops are markedly lower. This is also shown by the analyses of conditional relative survival, in which survival is only determined from the first, second, third and fourth year after diagnosis of MCC (Figure 2, Tables S3 and S4).

## 4. Discussion

The age-standardized incidence of MCC was 35.3% higher in men than in women. Each age group showed a higher incidence rate in men than in women. MCC of the skin of the head and neck was predominant in both men and women. The 5-year relative survival of MCC overall and in each age group was lower in men than it was in women. When comparing 5-year relative survival by anatomic location, it appears that the overall difference in survival from MCC between men and women is primarily due to differences in survival from MCC of the skin of the head and neck. It has previously been reported that the conditional survival of MCC patients is dynamic and increases with time since the initial diagnosis [30]. Our data confirm that the probability of survival after MCC diagnosis is most reduced in the first two years after diagnosis, and each additional year after diagnosis of MCC that is survived improves the probability of survival. This finding is important for the design and interpretation of adjuvant and neo-adjuvant therapeutic trials in MCC.

International comparisons of MCC incidence rates are complicated by differences in registration and evaluation methods, particularly the choice of age standard. A comparison of age-standardized rates (old European standard) in North Rhine-Westphalia, Germany (men: 5.2 per million person-years; women: 3.8 per million person-years), with France in 2006–2010 (men: 4.3 per million person-years; women 3.9 per million person-years) [31] and the Netherlands in 2016 (men: 6.7 per million person-years; women: 5.2 per million person-years) [11] shows that incidence rates are very similar in these countries, which mainly consist of non-Hispanic white populations and live at similar latitudes.

In our analyses, the 5yr-RS of men with MCC was 11.9% lower than that of women in our analyses. This is in line with other registry-based studies that reported 5yr-RS by sex. There was always a clear difference in survival to the detriment of men, including the Netherlands [10], Finland [12], Queensland [16], and Germany [15]—the only exception was one report from New Zealand [14]. To further investigate this sex effect, the authors of three of these papers examined the association between sex and survival using regression models adjusting for stage distribution, age, and anatomic site [11,14,16]. In all three studies, the sex effect persisted after adjustment, suggesting that regardless of the stage of MCC at diagnosis, men have a worse prognosis than women. Our multiple imputation analysis of observed overall and disease-specific survival that adjusted for age, anatomic location, and UICC stage corroborated this finding.

The sex difference in cancer survival have been reported before. Indeed, both incidence and survival of cancer vary markedly by sex, with males generally having lower survival compared with females. A pan-cancer analysis using data from the SEER program of 2001 to 2016 found that male-to-female survival differences were noted across various cancer types, races, and age groups [32]. A Swedish cohort study also reported that male sex is associated with increased risk and poorer survival for most cancer sites. Our findings add to the evidence that the fundamental biology of sex differences affects cancers of all types [33].

Two previous studies reported that the 5yr-RS in MCC is highest in the face or ears [14,16]. Neither of these two studies reported this finding in a sex-specific manner. Our sex-specific analyses showed that the 5yr-RS for MCC of the face or ears is high only in women (72.5%), while it is markedly lower in men (57.5%). We found the highest 5yr-RS for MCC of the upper limbs in both sexes.

In a recent publication, McEvoy et al. described one of the largest MCC cohorts of a university tertiary center for MCC in terms of clinical outcome and also provided a review of the published literature. In their cohort of 618 patients, they described an overall 5-year recurrence probability of 40% for all stages, with the majority of recurrences occurring within the first 2 years and 95% of recurrences occurring within the first 3 years [34]. Miura et al. found that the most critical interval in terms of survival of advanced-stage MCCs are the first two years after diagnosis [35]. We corroborated this finding through our detailed unconditional and conditional relative survival analysis of patients who survived the first, second, third, and fourth year after diagnosis of MCC.

Although we used a large data set of 2164 MCC patients, several limitations of our results exist. First, due to missing values for UICC stage, we had to use multiple imputation to replace missing values in order to adjust for the potentially confounding effect of tumor stage at initial diagnosis. Second, the important risk and prognosis factor of immunosuppression (e.g., due to hematologic neoplasia, HIV infection, or organ transplantation) is not available in the routine data of the cancer registries in Germany and could not be examined in detail [36]. Third, further morphological subtyping of MCC or establishing the MCPyV status was not possible as this would have required the detailed clinical description, detailed pathology findings, and additional molecular characterization of the specimens. While population-based cancer registries focus on epidemiologic measures of cancer, i.e., cancer incidence, its distribution by age, sex, and location of patients, and survival, specialized disease registries typically focus on very detailed phenotyping, including molecular pathology, precise treatment protocols including first-line, second-line, etc., detailed follow-up endpoints including progression, recurrence, and also patient-reported outcomes. The disadvantage of specialized disease registries, however, is often the lack of a clear population reference that would allow for the calculation of epidemiologic measures. Thus, in order to better combine both approaches, the German Cancer Early Detection and Registry Act (KFRG) came into force on 9 April 2013. While full implementation in all German states will require some time to establish the appropriate structures, a comparison of figures between specialized disease registries and population-based cancer registries over the last few years already shows the positive development, so that evaluations will soon be possible. Future high-resolution studies, i.e., in registries that also collect tissue samples, that allow for further biological subtyping of MCC and have detailed staging information may provide further insight into the differential prognosis of MCC in men and women.

## 5. Conclusions

Our data of a population-representative cancer registry not only provide up-to-date incidence and survival data for MCC and validate worse survival among men, but also highlight a more favorable prognosis for MCCs located on the limbs. Most crucially, however, we showed that the first two years after diagnosis of MCC are the years with the highest excess mortality of MCC patients. The latter is highly relevant for the design and interpretation of adjuvant and neo-adjuvant therapeutic trials in MCC.

## Figures and Tables

**Table 1:** Age-standardized incidence rate (cases per million person-years) of Merkel cell carcinoma among men and women in North Rhine-Westphalia, Germany, 2008–2021.

Characteristic	Men			Women		
	N	Rate	SE	N	Rate	SE
Overall	1049	5.2	0.16	1115	3.8	0.13
Localization						
Head	361	1.7	0.09	530	1.6	0.07
Face/Ear	274	1.2	0.08	486	1.5	0.07
Scalp/Neck	85	0.4	0.04	41	0.1	0.02
Trunk	114	0.6	0.06	63	0.3	0.04
Upper limb	276	1.4	0.09	236	0.9	0.06
Lower limb	144	0.8	0.06	153	0.6	0.05
Other	154	0.8	0.06	133	0.5	0.05
Age at diagnosis (years)						
<65	135	1.0	0.09	114	0.9	0.08
65–74	288	23.1	1.37	231	16.2	1.08
75–84	445	54.7	2.62	466	40.5	1.92
85+	181	96.1	7.14	304	68.4	3.92
UICC stages ^1^	N	%		N	%	
Non-missing	429			425		
I	185	43.1		216	50.8	
II	48	11.2		51	12.0	
III	91	21.2		76	17.9	
IV	55	12.8		34	8.0	
I–II	20	4.7		19	4.5	
III–IV	30	7.0		29	6.8	
Missing	620			690		

Rates are standardized by the “old” European standard population. SE: Standard error of the rate; ^1^ incidence rates for stages are not presented because of missing data.

**Table 2:** Five-year relative survival (%) of the years 2017–2021 among men and women with newly diagnosed Merkel cell carcinoma in North Rhine-Westphalia, Germany.

Characteristic	Men			Women		
	Patients (N)	Point Estimate %	95%CI	Patients (N)	Point Estimate %	95%CI
Overall	686	58.8	[52.3;65.4]	700	70.7	[64.2;77.3]
Localization						
Head and neck	233	54.0	[41.8;66.1]	350	72.5	[62.4;82.6]
Face/Ear	179	57.5	[43.5;71.5]	319	73.8	[63.2;84.4]
Scalp/Neck	52	43.2	[18.8;67.5]	29	59.7	[26.6;92.8]
Trunk	78	54.4	[36.3;72.5]	42	45.6	[24.3;67.0]
Upper limb	190	68.2	[55.8;80.6]	142	79.3	[65.8;92.8]
Lower limb	106	62.9	[46.4;79.3]	106	75.9	[60.9;90.9]
Other	79	48.1	[30.6;65.5]	60	49.8	[30.0;69.7]
Age at diagnosis (years)						
<65	95	65.8	[51.6;80.0]	73	78.4	[65.2;91.6]
65–74	179	62.3	[51.5;73.0]	152	81.7	[71.2;92.2]
75–84	297	56.4	[46.0;66.8]	295	69.8	[60.3;79.3]
85+	115	52.2	[28.7;75.8]	180	57.0	[39.3;74.7]

Point estimate: estimated 5-year relative survival by period approach in percentages; 95%CI: 95% confidence interval.

**Table 3:** Effect of sex on survival among patients with newly diagnosed Merkel cell carcinoma in North Rhine-Westphalia, Germany, 2008–2021.

Model	Cohort	Adjustment Set	Hazard Ratio	95%CI
Overall mortality				
#1	Complete cases (*n* = 854) ^1^	Empty	0.67	0.54–0.83
#2	Complete cases (*n* = 854) ^1^	Age, topography	0.58	0.46–0.72
#3	Complete cases (*n* = 854) ^1^	Age, topography, UICC	0.59	0.47–0.74
#4	Overall cohort (*n* = 2164)	Age, topography, UICC	0.69	0.61–0.78
Disease-specific mortality				
#5	Complete cases (*n* = 854) ^1^	Empty	0.58	0.40–0.83
#6	Complete cases (*n* = 854) ^1^	Age, topography	0.53	0.37–0.78
#7	Complete cases (*n* = 854) ^1^	Age, topography, UICC	0.55	0.38–0.81
#8	Overall cohort (*n* = 2164)	Age, topography, UICC	0.69	0.54–0.89

^1^ Complete cases (*n* = 854 cases) are a subset of the overall cohort with zero missing items regarding year, sex, topography, age at diagnosis, and UICC stage; the overall cohort (*n* = 2164) has zero missing items for these items after multiple imputation.

## Data Availability

The data can be requested from the Cancer Registry of North Rhine-Westphalia.

## Supplementary Materials

The following supporting information can be downloaded at: https://www.mdpi.com/article/10.3390/cancers16112158/s1, Figure S1: Data use for estimation of 5-year survival by period analysis for the calendar period 2017–2021 (solid frame, the numbers within the cells indicate the follow-up years after diagnosis that may be observed in the respective calendar year of follow-up); Table S1: Crude and age-standardized incidence rate (cases per million person-years) of Merkel cell carcinoma among men and women in North Rhine-Westphalia, Germany, 2008–2021; Table S2: Potential determinants of missing UICC stages among patients with newly diagnosed Merkel cell carcinoma in North Rhine-Westphalia, Germany, 2008–2021; Table S3: Unconditional and conditional relative survival (%) among men and women with newly diagnosed Merkel cell carcinoma in North Rhine-Westphalia, Germany, 2017–2021; Table S4: Absolute decline of survival (percentage points) from year to year among men and women with newly diagnosed Merkel cell carcinoma in North Rhine-Westphalia, Germany, 2017–2021. References [37,38,39] are cited in the Supplementary Materials.
