# Supplementary material for: Incidence and Relative Survival of Patients with Merkel Cell Carcinoma in North Rhine-Westphalia, Germany, 2008–2021
Source: Cancers (Basel). 2024 Jun 6;16(11):2158. doi: 10.3390/cancers16112158 (PMC11171844; doi:10.3390/cancers16112158)
Supplement: Supplementary file 1 [file cancers-16-02158-s001.zip › cancers-3040089-supplementary.pdf]

SUPPLEMENT

Incidence and relative survival of patients with Merkel cell carcinoma in North Rhine-Westphalia, Germany, 2008-2021

Andreas Stang,[1-3] Lennart Möller, Ina Wellmann,[3] Kevin Claaßen,[3] Hiltraud Kajüter,[3] Selma Ugurel,[4,7] Jürgen C. Becker[4-7]

Suppl. Figure S1

Data use for estimation of 5-year survival by period analysis for the calendar period 2017–2021 (solid frame, the numbers within the cells indicate the follow-up years after diagnosis that may be observed in the respective calendar year of follow-up)

Calendar year of diagnosis	Calendar year of follow-up									
	2012	2013	2014	2015	2016	2017	2018	2019	2020	2021
2012	1	1/2	2/3	3/4	4/5	5				
2013		1	1/2	2/3	3/4	4/5	5			
2014			1	1/2	2/3	3/4	4/5	5		
2015				1	1/2	2/3	3/4	4/5	5	
2016					1	1/2	2/3	3/4	4/5	5
2017						1	1/2	2/3	3/4	4/5
2018							1	1/2	2/3	3/4
2019								1	1/2	2/3
2020									1	1/2
2021										1

Suppl. Table S1

Crude and age-standardized incidence rate (cases per million person-years**) of Merkel cell carcinoma among men and women in North Rhine-Westphalia, Germany, 2008-2021**

Characteristic	Men			Women		
	N	Rate	SE	N	Rate	SE
**Crude**						
Overall	1049	8.6	0.27	1115	8.7	0.26
Localization						
Head and neck	361	3.0	0.16	530	4.2	0.18
Face/ear	274	2.2	0.14	486	3.8	0.17
Scalp/neck	85	0.7	0.08	41	0.3	0.05
Trunk	114	0.9	0.09	63	0.5	0.06
Upper limb	276	2.3	0.14	236	1.9	0.12
Lower limb	144	1.2	0.10	153	1.2	0.10
Other	154	1.3	0.10	133	1.0	0.09
Age at diagnosis (years)						
< 65	135	1.4	0.12	114	1.2	0.11
65-74	288	23.7	1.40	231	16.8	1.10
75-84	445	55.7	2.64	466	42.1	1.95
85+	181	96.1	7.14	304	68.4	3.92
**Age-standardized (World Standard Population)**						
Overall	1049	3.2	0.11	1115	2.3	0.08
Localization						
Head and neck	361	0.9	0.05	530	0.9	0.05
Face/ear	274	0.7	0.05	486	0.8	0.04
Scalp/neck	85	0.2	0.03	41	0.1	0.01
Trunk	114	0.4	0.04	63	0.2	0.03
Upper limb	276	0.9	0.06	236	0.6	0.04
Lower limb	144	0.5	0.04	153	0.4	0.04
Other	154	0.5	0.04	133	0.3	0.03
Age at diagnosis (years)						
< 65	135	0.8	0.07	114	0.6	0.06
65-74	288	22.8	1.36	231	16.0	1.07
75-84	445	54.7	2.62	466	40.5	1.92
85+	181	96.1	7.14	304	68.4	3.92

(Suppl. Table S1 continued)

Characteristic	Men			Women		
	N	Rate	SE	N	Rate	SE
**Age-standardized (U.S. 2000 Standard Population)**						
Overall	1049	6.3	0.20	1115	4.7	0.14
Localization						
Head and neck	361	2.3	0.12	530	2.1	0.09
Face/ear	274	1.7	0.11	486	1.9	0.09
Scalp/neck	85	0.5	0.06	41	0.2	0.03
Trunk	114	0.7	0.06	63	0.3	0.04
Upper limb	276	1.6	0.10	236	1.0	0.07
Lower limb	144	0.9	0.07	153	0.7	0.05
Other	154	0.9	0.07	133	0.6	0.05
Age at diagnosis (years)						
< 65	135	0.9	0.08	114	0.8	0.07
65-74	288	23.8	1.40	231	16.7	1.10
75-84	445	55.7	2.64	466	41.4	1.93
85+	181	96.1	7.14	304	68.4	3.92

Legend Table S1: SE: standard error of the crude rate;

Imputation model

We assumed a missing at random mechanism for staging information (UICC) and used several covariates to impute values for stage. Under the assumption of missing at random, multiple imputation corrects biases that may arise in complete case analyses (Sterne, 2009).

The imputation used the following categorical variables: UICC stage (I, II, III, IV, I-II, III-IV), anatomic subsite (head, trunk, upper limbs, lower limbs, and as a group overlapping, missing, genital or unknown subsites), sex (0, 1), vital status (survived, death due to Merkel cell carcinoma, unrelated death), and calendar year of diagnosis; age at diagnosis, estimated cumulative baseline hazard, and a multiplicative term for age and log (follow-up duration) were used as continuous variables. We approximated the cumulative baseline hazard using the Nelson-Aalen estimator (White & Royston, 2009). We ran 20 burn-in iterations before 20 multiple imputations and used the discriminant function method of classification variables. We used an noninformative prior. In a sensitivity analysis, we performed the imputation of UICC stages based on T, N and M information instead of UICC stages. The results were almost identical (results not shown).

Analysis model

The analysis models were Cox proportional hazards regressions for the endpoints overall mortality and disease-specific mortality including the following class variables: UICC-stage, sex, age group, and anatomic subsite. All variables were included as categorical variables in the model. The effect estimates of the 20 imputed data sets were pooled by Rubin's rule (Rubin, 1987) using using SAS PROC MIANALYZE. All analyses were done with SAS 9.4 (Cary, NC, USA).

References

RubinDB. Multiple imputation for nonresponse in surveys. NewYork:Wiley; 1987.

Sterne JAC. Multiple imputation for missing data in epidemiological and clinical research: potential and pitfalls. BMJ 2009;338:b2393

White IR, Royston P. Imputing missing covariate values for the Cox model. Stat Med 2009;28:1982e98.

Suppl. Table S2

Potential determinants of missing UICC stages among patients with newly diagnosed Merkel cell carcinoma in North Rhine-Westphalia, Germany, 2008-2021

Characteristic	Patients without missing UICC stage		Patients with missing UICC stage		Association between baseline data and missing UICC stage	
	N	%	N	%	PR	95%CI
Sex						
Men	429	40.9	620	59.1	Ref.	
Women	425	38.1	690	61.9	1.05	0.98-1.12
Age at diagnosis (years)						
< 60	63	48.8	66	51.2	Ref.	
60-69	147	45.8	174	54.2	1.06	0.87-1.29
70-79	311	40.0	466	60.0	1.17	0.98-1.40
80-89	299	37.6	497	62.4	1.22	1.02-1.46
≥ 90	34	24.1	107	75.9	1.48	1.22-1.80
Anatomic localization						
Head	367	41.2	524	58.6	Ref.	
Trunk	70	39.6	107	60.5	1.03	0.90-1.17
Arms	217	42.4	295	57.6	0.98	0.89-1.07
Legs	144	48.5	153	51.5	0.88	0.77-0.99
Overlapping, missing, or genital skin	56	19.5	231	80.5	1.37	1.26-1.48
Year of diagnosis						
2008	16	14.8	92	85.2	Ref.	
2009	14	12.3	100	87.7	1.03	0.93-1.14
2010	19	15.0	108	85.0	1.00	0.90-1.11
2011	36	22.2	126	77.8	0.91	0.81-1.02
2012	34	25.2	101	74.8	0.88	0.77-1.00
2013	47	30.3	108	69.7	0.82	0.72-0.93
2014	59	36.0	105	64.0	0.75	0.65-0.86
2015	47	27.8	122	72.2	0.85	0.75-0.96
2016	73	42.9	97	57.1	0.67	0.58-0.78
2017	97	52.4	88	47.6	0.56	0.47-0.66
2018	78	46.7	89	53.3	0.63	0.53-0.74
2019	97	55.4	78	44.6	0.52	0.44-0.63
2020	103	65.6	54	34.4	0.40	0.32-0.51
2021	134	76.1	42	23.9	0.28	0.21-0.37
Death						
No	511	49.4	523	50.6	Ref.	
Yes	343	30.4	787	69.6	1.38	1.28-1.48
Due to MCC	121	40.7	176	59.3	1.17	1.05-1.31
Unrelated	222	26.7	611	73.3	1.45	1.35-1.56

Legend Suppl Table S2: PR: prevalence ratio, i.e., the ratio of the percentage of missing data by study characteristic; Ref: reference group; 95%CI: 95% confidence interval; a prevalence ratio of e.g. 0.28 in 2021 means that the probability of missing UICC stage in this year was 72% lower than in 2008; UICC: Union internationale contre le cancer;

Suppl. Table S3 **Unconditional and conditional relative survival (%) among men and women with newly diagnosed Merkel cell carcinoma in North Rhine-Westphalia, Germany, 2017-2021**

	Year of follow-up										
**Conditioning**	**0**	**1**		**2**		**3**		**4**		**5**	
**Men**											
0	100	87.0	(1.94)	73.8	(2.59)	67.6	(2.89)	62.0	(3.13)	58.8	(3.36)
1		100		84.8	(2.30)	77.8	(2.84)	71.3	(3.23)	67.6	(3.55)
2				100		91.6	(2.24)	84.0	(3.06)	79.7	(3.59)
3						100		91.7	(2.46)	87.0	(3.28)
4								100		94.9	(2.52)
**Women**											
0	100	87.3	(2.01)	80.4	(2.52)	76.7	(2.83)	73.3	(3.1)	70.7	(3.34)
1		100		92.1	(1.95)	87.9	(2.53)	84.0	(2.98)	81.1	(3.34)
2				100		95.4	(1.87)	91.2	(2.59)	88.0	(3.11)
3						100		95.6	(1.97)	92.2	(2.71)
4								100		96.5	(2.02)

Legend Suppl Table S3: Conditioning 0 means that relative survival is estimated from the date of diagnosis; conditioning 1 means that relative survival is estimated starting after 1-year survival of MCC and so on; in parentheses: SE - standard error of the relative survival estimate; all relative survival estimates are based on the period analysis.

Suppl. Table S4 **Absolute decline of survival (percentage points) from year to year among men and women with newly diagnosed Merkel cell carcinoma in North Rhine-Westphalia, Germany, 2017-2021**

	Year of follow-up: yearly drop of survival (percentage points)				
**Conditioning**	0 → 1	1 → 2	2 → 3	3 → 4	4 → 5
**Men**					
0	13.0	13.2	6.2	5.6	3.2
1		15.2	7.0	6.5	3.7
2			8.4	7.6	4.3
3				8.3	4.7
4					5.1
**Women**					
0	12.7	6.9	3.7	3.4	2.6
1		7.9	4.2	3.9	2.9
2			4.6	4.2	3.2
3				4.4	3.4
4					3.5

Legend Suppl Table S4: Conditioning 0 means that relative survival is estimated from the date of diagnosis; conditioning 1 means that relative survival is estimated starting after 1-year survival and so on; 0 → 1 means survival from date of diagnosis up to the end of the first year; 1 → 2 means survival within the second year after diagnosis and so on; all relative survival estimates are based on the period analysis.
